# GLP-1 secretion by microglial cells and decreased CNS expression in obesity

**DOI:** 10.1186/1742-2094-9-276

**Published:** 2012-12-23

**Authors:** Camilla Kappe, Linda M Tracy, Cesare Patrone, Kerstin Iverfeldt, Åke Sjöholm

**Affiliations:** 1 Karolinska Institutet, Department of Clinical Science and Education, Södersjukhuset, Stockholm, 11883, Sweden; 2 Department of Neurochemistry, Stockholm University, Stockholm, 10691, Sweden

**Keywords:** Glucagon-like peptide-1, Microglia, Neuroinflammation, Neuroprotection, Proglucagon

## Abstract

**Background:**

Type 2 diabetes (T2D) is a strong risk factor for developing neurodegenerative pathologies. T2D patients have a deficiency in the intestinal incretin hormone GLP-1, which has been shown to exert neuroprotective and anti-inflammatory properties in the brain.

**Methods:**

Here we investigate potential sources of GLP-1 in the CNS and the effect of diabetic conditions on the proglucagon mRNA expression in the CNS. The obese mouse model *ob/ob*, characterized by its high levels of free fatty acids, and the microglia cell line BV-2 were used as models. mRNA expression and protein secretion were analyzed by qPCR, immunofluorescence and ELISA.

**Results:**

We show evidence for microglia as a central source of GLP-1 secretion. Furthermore, we observed that expression and secretion are stimulated by cAMP and dependent on microglial activation state. We also show that insulin-resistant conditions reduce the central mRNA expression of proglucagon.

**Conclusion:**

The findings that microglial mRNA expression of proglucagon and GLP-1 protein expression are affected by high levels of free fatty acids and that both mRNA expression levels of proglucagon and secretion levels of GLP-1 are affected by inflammatory stimuli could be of pathogenic importance for the premature neurodegeneration and cognitive decline commonly seen in T2D patients, and they may also be harnessed to advantage in therapeutic efforts to prevent or treat such disorders.

## Background

Type 2 diabetes (T2D) has been identified as a risk factor for developing neurodegenerative pathologies, such as Alzheimer’s disease (AD), and stroke [1-3]. Premature cognitive decline is also a common feature in T2D patients. Although it has been speculated that chronic hyperglycemia or recurrent hypoglycemia may be involved in these derangements, the precise nature of the underlying mechanisms remain elusive. T2D patients are not only characterized by hyperglycemia, but typically also have increased circulating levels of free fatty acids that may accumulate in the central nervous system (CNS) [4].

Incretin hormones, such as glucagon-like peptide-1 (GLP-1), are derived from proglucagon, which is produced by enteroendocrine L-cells, and augment meal-stimulated insulin secretion in a glucose-dependent manner [5]. In T2D, the incretin response has been reported to be impaired as a result of defective GLP-1 secretion [6]. In addition to stimulating insulin secretion, GLP-1 exerts CNS actions such as neuroprotection and reduced appetite [7,8] that appear independent of its glycemic effects. Increasing evidence suggests that GLP-1 and its analogs play a very important neuroprotective role [9]. In GLP-1R knockout mice, neuronal injury is increased after kainate administration [2,10], and GLP-1 has been shown to protect the neuroblastoma cell line SH-SY5Y from amyloid-β-induced apoptosis [11]. There is also evidence that the GLP-1 mimetic exendin-4, clinically used against T2D, improves cognitive performance [2] and stimulates neurogenesis [12]. In fact, the National Institute on Aging (NIA) is currently recruiting for a clinical trial with exendin-4 for the treatment of AD. In addition, GLP-1 is well known to have anti-inflammatory properties in different tissues [13]. Considering the importance of GLP-1 in both T2D and neurodegenerative pathologies, and the strong co-morbidity between T2D and different neurodegenerative pathologies, the neuroprotective role and nature of GLP-1 secretion and action in the brain have become hot topics. Further, entirely consistent with an important neuroprotective role of GLP-1, the GLP-1R is abundantly expressed in many parts of the brain, including the hypothalamus, hippocampus and cortex. In contrast, the only known source of GLP-1 secretion in the CNS to date is the *nucleus tractus solitarius* (NTS) neurons of the brainstem [14], although Iwai *et al.* reported the presence of GLP-1 immunoreactive material in cultured rat microglia [15]. Here, we aimed to investigate whether there in fact are non-neuronal sources of GLP-1 secretion in the brain, and, if so, how secretion of GLP-1 can be stimulated and how it is affected by obesity and insulin-resistant conditions. Such knowledge may shed light on the pathogenesis of neurodegeneration in T2D patients. Ultimately, this information may also lead to novel ways of stimulating neuroprotection in conditions linked to neurodegeneration, such as T2D, by modulating the secretion of one of the brain’s own neuroprotective peptides.

## Materials and methods

### Animals

Obese (*ob/ob*) and lean (+/+) male mice were obtained from our breeding colony of C57BL/6 J-*ob/+* mice. The breeding colony was housed at 23°C, and the mother was removed from the pups when they were 3 weeks of age. At 3.5 weeks of age, obese and lean pairs were separated from their littermates and were housed individually in solid-bottom, plastic cages with wood shavings and paper as bedding. The experimental procedures were approved by Stockholm South local committee on ethics of animal experiments and performed in accordance with international standards on animal welfare. All mice had food and water available *ad libitum*. At 6-9 months of age, animals were sacrificed and the brains were removed and cortex and hippocampus dissected. These brain tissues were analyzed together. Alternatively, the brains were used for preparation of tissue sections.

### Cell culture and reagents

The BV-2 cell line [16] was cultured in DMEM Glutamax supplemented with 5% fetal bovine serum, 10,000 U/ml penicillin and 10 mg/ml streptomycin sulfate under 5% CO_2_ at 37°C.

Palmitate (sodium palmitate, Sigma-Aldrich, St. Louis, MO) exposure medium was supplemented with 0.5% bovine serum albumin (BSA, fatty acid free; Sigma-Aldrich). Palmitate was dissolved in 12.5% ethanol during heating to 60°C. Control cells were given vehicle with equal amounts of ethanol as the palmitate-exposed cells. Other reagents used included Forskolin (Sigma-Aldrich) and lipopolysaccharide (LPS) (Sigma-Aldrich).

Primary rat glial cells cultures consisting of 5-10% microglia and 90-95% astrocytes were isolated from the cortex of Sprague-Dawley pups less than 24 h of age as previously described [17]. Cells were seeded in 60-mm culture dishes and maintained in DMEM Glutamax containing 10% fetal bovine serum (FBS) and 0.1% penicillin-streptomycin (PEST) for 20 days (all from GIBCO). Medium was exchanged every 3-4 day.

### Preparation of tissue sections

Isolated mouse brains were placed in 4% paraformaldehyde (PFA) (Sigma-Aldrich) and fixated overnight at 4°C. The fixed tissue was then placed in 30% sucrose and stored in 30% sucrose at 4°C. Prior to sectioning the tissue, the stage of the cryocutter was cooled by addition of dry ice. The tissue was mounted on the cold stage using tissue tech (Histolab, Göteborg, Sweden) and covered in dry ice long enough to allow the tissue to take a frozen form. The first few millimeters of tissue was removed, and then 40-μm-thick coronal sections were cut, using a sliding microtome, throughout the whole brain and placed in antifreeze prior to storage in -30°C.

### Immunohistochemistry/immunocytochemistry

#### Chromogenic visualization using diaminobenzidine

Tissue sections were washed in phosphate-buffered saline (PBS) 3 × 5 min, quenched in hydrogen peroxide for 15 min, before being washed again in phosphate buffer containing 0.25% Triton X-100 (PBS-T) 3 × 5 min. Sections were then incubated overnight at 4°C with the primary GLP-1-specific antibody (Phoenix peptides) at 1:50 dilution. The primary antibody was detected by use of biotin-conjugated (Vector) secondary antibody (1:200 dilution). Sections were incubated with the secondary antibody for 2 h at room temperature (approximately 21°C) in phosphate buffer containing 5% of the appropriate serum and 0.25% Triton X-100. Following this, a 1-h incubation at room temperature with avidin–biotin complex (ABC kit; Vector) was allowed before subsequent addition of diaminobenzidine.

#### Fluorescent visualization

Tissue sections were washed in PBS-T 3 × 5 min, blocked in 5% milk and incubated overnight at 4°C with primary antibodies (1:50 dilution in PBS-T containing 5% of the appropriate serum); GLP-1 (Phoenix peptides) and CD11b (AbDSerotec, Raleigh, NC). Sections were washed and incubated at room temperature for 1 h in the dark with the secondary antibodies (1:1,000 and 1:2,000 dilution in PBS-T), ALEXA Fluor 488 donkey anti-rabbit and ALEXA Fluor 568 goat anti-mouse (Invitrogen). The sections were washed again and mounted on glass slides using a soft brush to pick them out of the solution. Sections were allowed to dry in the dark before mounting the cover slip using mounting medium (Histolab).

Primary cells were cultured on a cover glass, washed with pre-warmed PBS-T and fixed with ice-cold 4% PFA for 10 min at room temperature. Cells were washed with PBS-T and incubated with primary antibodies (1:50 dilution in PBS-T containing 5% of the appropriate serum), GLP-1 (Phoenix peptides) and CD11b (AbDSerotec). Cells were washed with 5% milk solution and incubated at room temperature for 1 h (kept dark) with the secondary antibodies (1:1,000 and 1:2,000 dilution in PBS-T), ALEXA Fluor 488 donkey anti-rabbit and ALEXA Fluor 568 goat anti-mouse (Invitrogen). The cells were washed again and incubated for 1 min with DAPI (Invitrogen) prior to a final wash with PBS. The cover slips with cells were then mounted upside down on glass slides using mounting medium (Histolab).

### GLP-1 secretion assays

For palmitate treatment, BV-2 cells were plated at a density of 15,000 cells/cm^2^ and grown in 24-well plates for 24 h. Cells were then treated with 0.125 mM palmitate or vehicle for an additional 24 h. Immediately after the 24 h incubation, medium was collected and DPP-4 inhibitor added (10 μl/ml) (Millipore Corp., Billerica, MA) to prevent GLP-1 degradation. Samples were stored at -70°C pending ELISA.

For acute stimulation with agents, BV-2 cells were plated at a density of 75,000 cells/cm^2^ and grown in 24-well plates for 24 h. The medium was discarded, and the cells were washed with pre-warmed Krebs Ringer buffer (KRBH) buffer/0.2% BSA/0 mM glucose, followed by a 30-min pre-incubation with the same buffer. Cells were then treated with LPS (1 μg/ml) or the adenylyl cyclase activator forskolin (10 μM) for 2 h. Immediately thereafter, DPP-4 inhibitor was added (10 μl/ml), and the buffer was collected. Samples were stored at -70°C pending ELISA.

GLP-1 ELISA (Millipore Corp.) was performed according to the manufacturer’s instructions. This ELISA is specific for the bioactive forms of GLP-1 [GLP-1 (7-36) amide and GLP-1 (7-37)] and will not detect other forms of GLP-1. There is no crossreactivity with glucagon. All experiments were performed in triplicates and repeated ≥ three times to assess consistency of results.

### TNFα secretion assay

BV-2 cells were plated at a density of 15,000 cells/cm^2^ and grown for 24 h prior to exposure to 0.125 mM palmitate for 24 h or 1 μg/ml LPS for 2 h. The culture medium was collected and analyzed for TNFα on a solid phase ELISA kit (R&D Systems, Abingdon, UK) according to the manufacturer’s protocol.

### RNA extraction, cDNA synthesis and quantitative RT-PCR

BV-2 cells or animal tissue were lysed and RNA extracted using Aurum total RNA mini kit (catalog no. 7326820; BioRad Laboratories) according to the manufacturer’s instructions. cDNA was synthesized for qPCR using iScript™ cDNA synthesis kit (BioRad Laboratories) according to the manufacturer’s instructions.

For qPCR, glyceraldehyde-3-phosphate dehydrogenase (GAPDH) mRNA expression was used as an internal control. GAPDH is one of the most commonly used housekeeping genes for comparisons of gene expression data.

For each sample, the mRNA level of each target gene relative to *GAPDH* was estimated by calculating the DeltaCt or ΔCt (Ct_Target_ Gene − Ct_GAPDH_) and then converting to 2^−ΔCt^. To compare mRNA levels between experimental groups, the ratio of the average 2 ^−ΔCt^ for each treatment group relative to the control group (2^−ΔΔCt^) was determined for each gene. This ratio represents a fold change for each gene. Plotted as arbitrary units are the values generated for fold change. Differences in the scales/size of arbitrary units between the different analyses (see Figure 2) stem from the use of samples from different animals in the different groups skewing the average ΔCt of the groups and thus the fold change (2^−ΔΔCt^).

GAPDH primers were designed according to: ATGACATCAAGAAGGTGGTG, TGTCATACCAGGAAATGAGC using Invitrogen custom primer design software (Invitrogen, Inc.).

Proglucagon primers were designed according to: GATTTTGTGCAGTGGTTGAT, ACTTCTTCTGGGAAGTCTTCG using Invitrogen custom primer design software (Invitrogen, Inc.).

TNFα primers were designed according to: AAAGTCAACCTCCTCTCTGC, GGACTCCGCAAAGTCTAAGT using Invitrogen custom primer design software (Invitrogen, Inc.).

Interleukin-1β primers were designed according to: CTTTTCGTGAATGAGCAGAC, GAGGAAAACACAGGCTCTCT using Invitrogen custom primer design software (Invitrogen, Inc.).

Arginase 1 primers were designed according to: TATGTGTCATTTGGGTGGAT, GCCAATGTACACGATGTCTT using Invitrogen custom primer design software (Invitrogen, Inc.).

Chitinase-3 primers were designed according to: GACTTGCGTGACTATGAAGC, TGACGGTTCTGAGGAGTAGA using Invitrogen custom primer design software (Invitrogen, Inc.).

A one-step RT-PCR kit with SYBR Green (iScript™ one-step RT-PCR kit with SYBR® Green) (BioRad Laboratories) was used for real-time quantitative RT-PCR. This kit utilizes iScript RNase H + reverse transcriptase and hot-start iTaq DNA polymerase. GAPDH was used as a housekeeping gene for normalization.

### Statistical analysis

Comparisons between control and single treatment groups with normalized distribution of data were done using two-tailed Student’s *t* test. In the case that normality tests failed, Mann-Whitney rank sum test was used for comparisons between the control and single treatment group. Correlations were evaluated by determining the Pearson correlation coefficients. *P* < 0.05 was deemed statistically significant.

## Results

### GLP-1-positive cells are detected in cerebral cortex of mice and significantly reduced in an obese insulin-resistant mouse model

**Figure 1:**
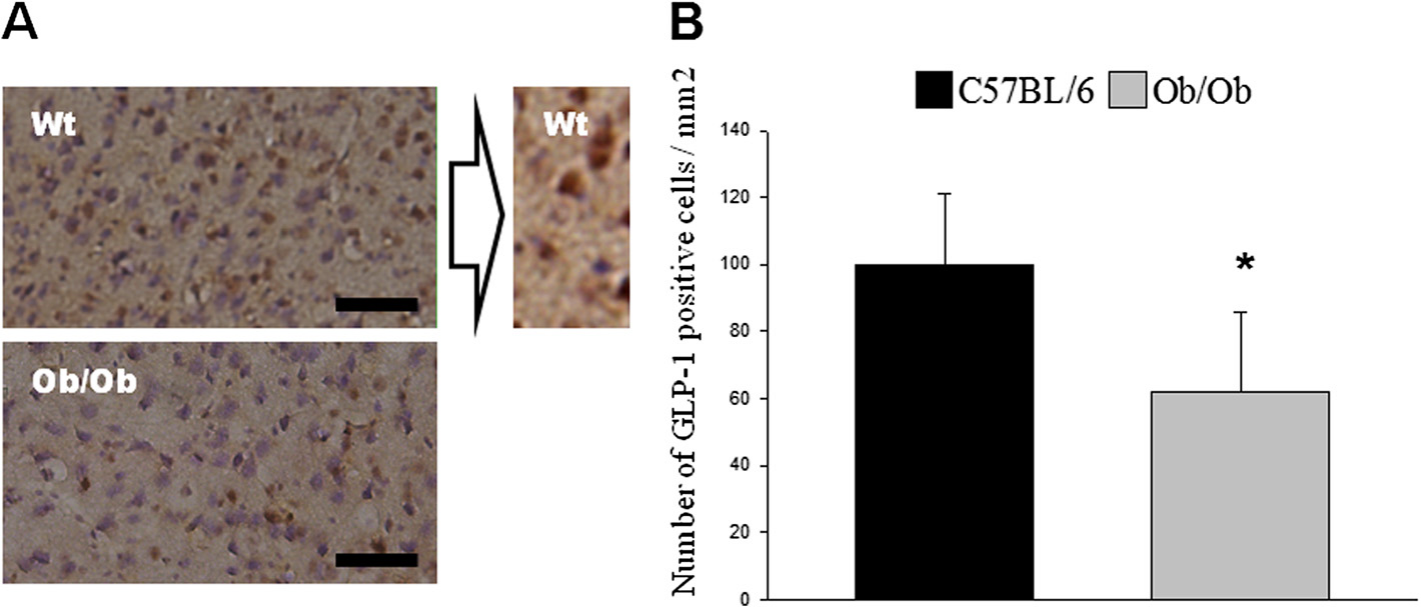
**The number of GLP-1-positive cells in the frontal cortex is reduced in an obese insulin-resistant mouse model. A**. Representative images from the frontal cortex adjacent to the hippocampus of 36-week-old mice showing relative abundance of GLP-1-positive cells (brown) in C57 black control animals (top left panel) —increased magnification (100 X) of GLP-1-positive cells (top right panel) —as compared to obese insulin-resistant *ob/ob* mice (bottom panel). Scale bar: 80 μm. **B**. Statistical analysis showing a significant decrease in the number of GLP-1-positive cells/area—in the frontal cortex adjacent to the hippocampus—in obese insulin-resistant *ob/ob* mice as compared to healthy controls. Data from 4 C57 black mice and 4 *ob/ob* mice. Bars represent mean ± SEM. * *P* < 0.05 for a chance difference *vs.* C57 black mice. Statistical analysis was performed using Mann-Whitney rank sum test.

To achieve the aim of this study, we investigated whether GLP-1-positive cells could be detected in parts of the brain outside of the nucleus of the solitary tract, where proglucagon mRNA expression has been previously observed. Results show that GLP-1-positive cells were indeed detected in cortex and hippocampus of adult C57 black and *ob/ob* male mice (Figure 1A). In addition, the number of GLP-1-positive cells was significantly reduced in the obese insulin-resistant *ob/ob* mouse model as compared to normal C57 black mice (Figure 1B).

### Proglucagon mRNA expression is detected in cerebral cortex and hippocampus of mice and significantly reduced in an obese insulin-resistant mouse model, while also strongly correlated to the mRNA expression of the proinflammatory tumor necrosis factor-α (TNFα)

**Figure 2:**
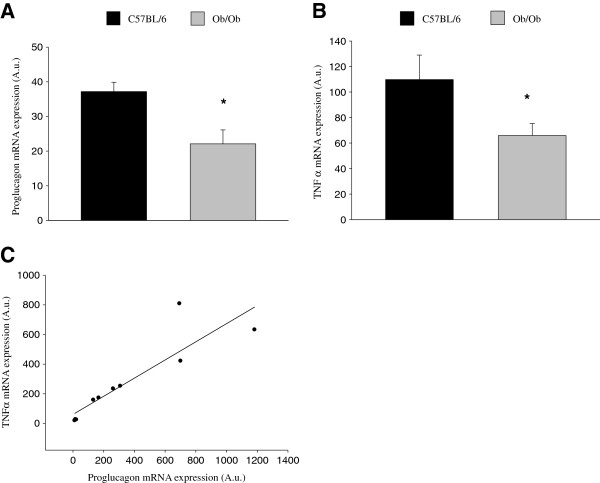
**CNS proglucagon mRNA expression in the frontal cortex is reduced in an obese insulin-resistant mouse model. A**. Proglucagon mRNA expression in obese *ob/ob* mice as compared to C57 black littermates. Data are from two groups of adult mice, in total 6 C57 black mice and 9 *ob/ob* mice, 6-9 months of age. **B**. TNFα mRNA expression in obese *ob/ob* mice as compared to C57 black littermates. Data from the same animals as in **A**. Proglucagon and TNFα mRNA expression was normalized with mRNA expression of GAPDH, which was used as an internal control. Bars represent mean ± SEM. *P < 0.05 for a chance difference. Statistical analysis was performed using Mann-Whitney rank sum test. **C**. Correlation between proglucagon mRNA and TNFα mRNA. Data from three C57 black mice and five *ob/ob* mice, 6 months of age; qPCR run in duplicate at two independent occasions. A.u.; arbitrary units.

To further confirm our finding of GLP-1-positive cells in an area outside of the nucleus of the solitary tract, where proglucagon mRNA expression has been previously observed, we investigated if proglucagon gene expression could be detected. Results show that proglucagon mRNA expression was indeed detected in the cortex and hippocampus of adult C57 black and *ob/ob* male mice (Figure 2A). In addition, the expression of the proglucagon gene was significantly reduced in the obese insulin-resistant *ob/ob* mouse model as compared to normal C57 black mice (Figure 2A). Since low-grade systemic inflammation is recognized in T2D [18], we also investigated if the expression of the proglucagon gene was correlated to the presence of inflammation in these areas of the brain. Thus, we determined the mRNA expression of the inflammatory markers TNFα and interleukin-1β (IL-1β) in the same tissue samples. Results show that, whereas no significant difference in the mRNA expression of IL-1β (115 ± 15% of expression in C57 black) could be detected between C57 black and *ob/ob* mice, significantly lower levels of TNFα mRNA expression were detected in *ob/ob* mice (Figure 2B). Further, there was a strong positive correlation between proglucagon mRNA expression and the mRNA expression of TNFα (Figure 2C).

### Microglial cells express the proglucagon gene and secrete GLP-1 in a cAMP-dependent manner

**Figure 3:**
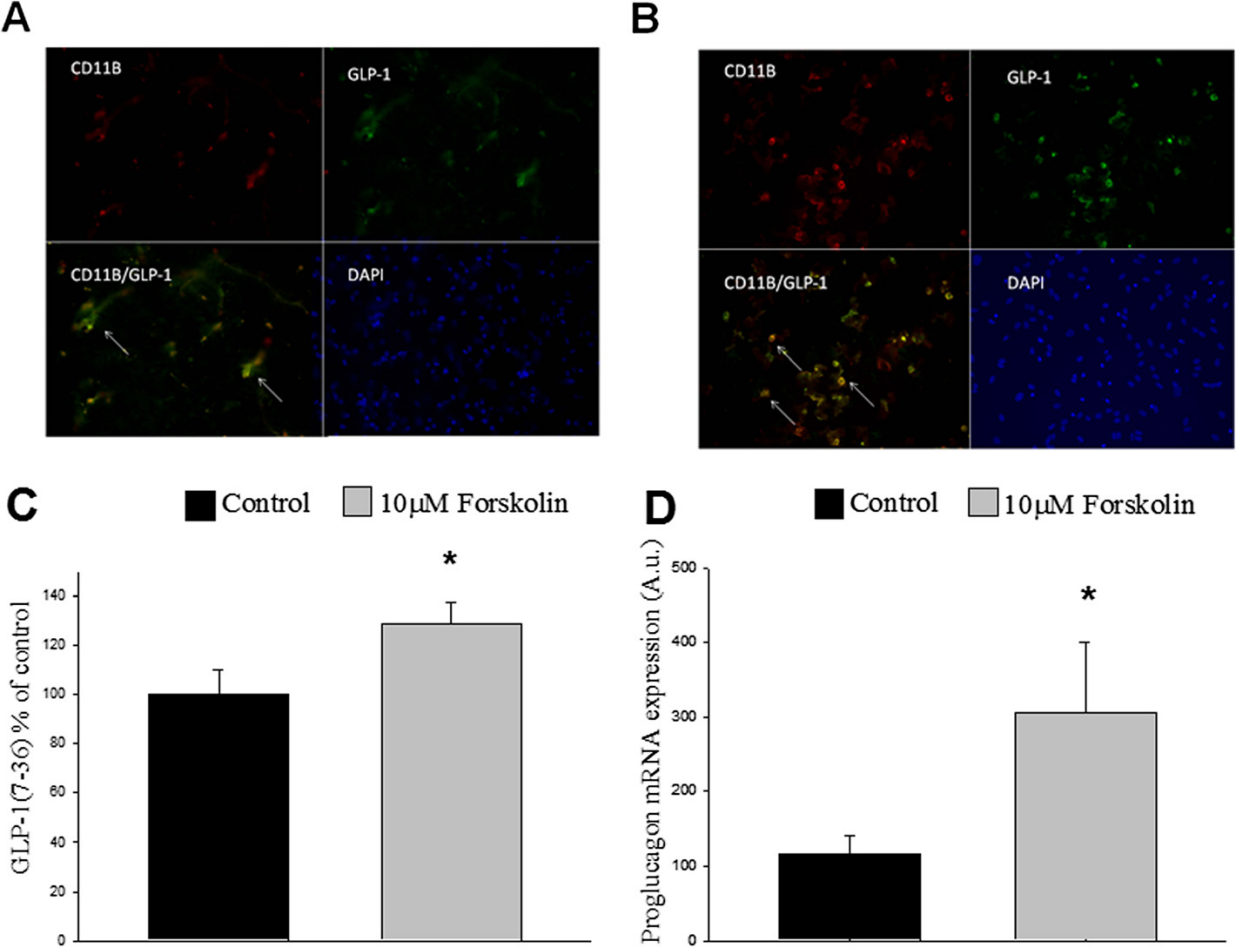
**Microglia express proglucagon mRNA and express and secrete GLP-1.** GLP-1-positive cells in **A**, tissue sections from the murine cortex, and **B**, primary glial cell cultures, GLP-1-positive (green, top right panel) microglia (red, top left panel). Cells positive for GLP-1 are also positive for the microglia marker CD11b (bottom left panel), whereas the remaining glial cells in the culture (indicated by blue DAPI staining in the bottom right panel) are positive for neither GLP-1 nor CD11b. White arrows indicate cells double-positive for CD11b and GLP-1. **C**. BV-2 cells secrete GLP-1, and this secretion is stimulated in response to a 2-h treatment with 10 μM of the adenylyl cyclase activator forskolin. GLP-1 secretion was measured as pM and presented as percent of control. **D**. Expression of proglucagon is increased in response to a 2-h treatment with 10 μM forskolin. Proglucagon mRNA expression was normalized to mRNA expression of GAPDH, which was used as an internal control. Bars represent mean ± SEM, *n* = 3-4, in triplicates. * *P* < 0.05 for a chance difference *vs.* untreated controls. Statistical analysis was performed using Student’s *t*-test.

Since neurons in cerebral cortex and hippocampus have been reported to lack expression of proglucagon [19], and after observing the strong correlation of proglucagon mRNA expression in these areas to the expression of the inflammatory marker TNFα, we investigated resident immune cells of the CNS (microglia) as a possible source of the proglucagon mRNA expression observed in these areas of the brain. Co-staining of tissue sections from the cortex for the CD11b antigen expressed on monocytes/macrophages and microglia, and GLP-1, revealed GLP-1-positive microglia in the murine cortex (Figure 3A). We also investigated GLP-1 expression in primary rat mixed glial cells and detected GLP-1-positive microglia cells (Figure 3B). To further confirm microglia as a source of proglucagon mRNA expression, as well as regulated GLP-1 secretion, we used the murine microglia BV-2 cell line. We found that these microglia-derived cells indeed express the proglucagon gene and secrete GLP-1 (Figure 3C,D). Previously, cyclic AMP has been shown to stimulate proglucagon gene transcription and GLP-1 production in other cell types [20]. As expected, in microglial cells both mRNA expression of the proglucagon gene and GLP-1 secretion were enhanced by adenylyl cyclase activation induced by forskolin (Figure 3C,D).

### GLP-1 secretion from microglial cells is regulated by inflammation

**Figure 4:**
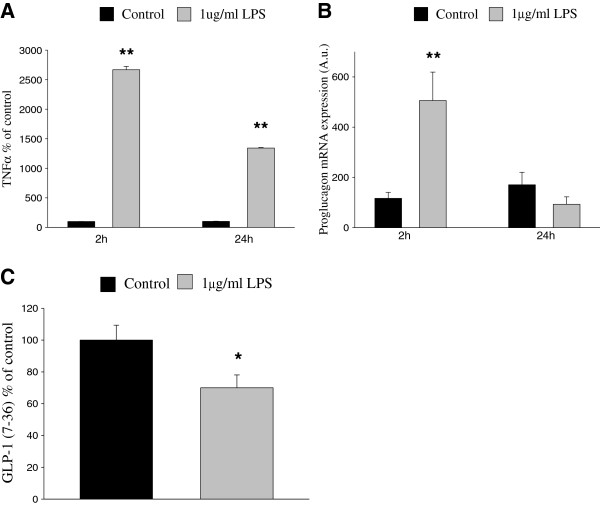
**Microglial GLP-1 secretion is decreased in response to inflammatory stimuli.A**. TNFα secretion is increased in response to 2-h and 24-h LPS treatment. TNFα secretion was measured as pg/ml and presented as percent of control. **B**. Expression of proglucagon is transiently increased in response to a 2-h treatment with LPS, with a clear trend toward reduced levels after 24 h. Proglucagon mRNA expression was normalized to mRNA expression of GAPDH, which was used as an internal control. **C**. Secretion of GLP-1 from BV-2 cells is inhibited by a 2-h treatment with 1 μg/ml LPS. GLP-1 secretion was measured as pM and presented as percent of control. Bars represent mean ± SEM, *n* = 3-4, in triplicates. * *P* < 0.05 for a chance difference *vs.* untreated controls. Statistical analysis was performed using Mann-Whitney rank sum test (**A,B**) and Student’s *t*-test (**C**).

In agreement with previous studies [21], classical activation of BV-2 microglial cells with lipopolysaccharide (LPS) increases the secretion of TNFα (Figure 4A). We report here that acute (2 h) activation of BV-2 cells with LPS also transiently increases proglucagon mRNA expression, while there is a clear trend toward reduced proglucagon expression after 24 h (Figure 4B). However, the increased mRNA expression after 2 h occurs in conjunction with a significant decrease in the amount of GLP-1 secreted from these cells (Figure 4C).

### Proglucagon mRNA expression is decreased in alternatively activated microglial cells in an in vitro model of T2D

**Figure 5:**
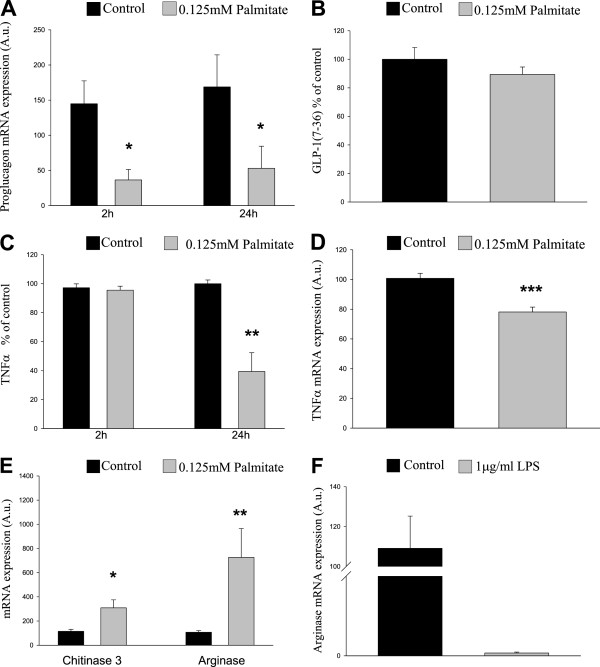
**Proglucagon gene expression in microglia is decreased by the free fatty acid palmitate.** Microglia were exposed to 0.125 mM palmitate for 24 h. **A**. Proglucagon gene expression is significantly reduced in microglia exposed to palmitate (mimicking the diabetic milieu in vitro). **B**. GLP-1 secretion is not significantly reduced after treatment of microglia with palmitate. GLP-1 secretion was measured as pM and presented as percent of control. **C**. No significant effect on TNFα secretion is detected after a 2-h exposure to palmitate; however, TNFα secretion is reduced from microglia treated with palmitate for 24 h. TNFα secretion was measured as pg/ml and presented as percent of control. **D**. TNFα expression is significantly reduced in microglia exposed to palmitate. **E**. Treatment with palmitate increases the expression of chitinase-3 and arginase 1. **F**. Treatment with LPS decreases the expression of arginase 1 after 24 h. mRNA expression was calculated after normalizing to mRNA expression of GAPDH, used as an internal control. Bars represent mean ± SEM, *n* = 3, in triplicates. * and **P < 0.05 and *P* < 0.001, respectively, for chance differences *vs.* untreated controls. Statistical analysis was performed using Mann-Whitney rank sum test and Student’s *t*-test (**C**,**D**).

Since proglucagon mRNA expression was reduced in the cortex and hippocampus of *ob/ob* mice (Figure 1A), we aimed to determine the effects of diabetic conditions on GLP-1 secretion from microglial cells in vitro*.* To simulate diabetic conditions, we cultured the BV-2 microglial cells in the presence or absence of a high concentration (0.125 mM) of the saturated fatty acid palmitate and determined the amount of GLP-1 secreted into the medium, as well as proglucagon mRNA expression levels. Our results demonstrate that high levels of fatty acid significantly reduced proglucagon mRNA expression after 2 h, as well as after 24 h. However, no significant effect on GLP-1 secretion could be detected in response to simulated hyperlipidemia (24-h incubation with palmitate) (Figure 5A,B). Since a strong positive correlation between proglucagon and TNFα mRNA expression was detected *in vivo* (Figure 1C), the effect of high levels of palmitate on TNFα mRNA expression and secretion from the microglial cells was determined. In line with the *in vivo* results, palmitate significantly reduced TNFα secretion and mRNA expression after 24 h (Figure 5C,D). To verify that these effects were not the result of a toxic effect of palmitate, we measured protein content in the wells at the end of the 24-h incubation. Our data demonstrate that palmitate does not alter the amount of protein in the wells after 24 h (control: 3.90 ± 0.29 μg/μl *vs.* palmitate: 3.65 ± 0.35 μg/μl). The decrease in TNFα mRNA expression and secretion in response to simulated hyperlipidemia (24 h incubation with palmitate) is unexpected, considering the systemic inflammation in T2D and that TNFα secretion increases in response to a classical activation of microglia cells. This prompted us to investigate a possible alternative activation of the microglial cells in response to palmitate. As shown in Figure 5E, we found that hyperlipidemia simulated by a 24-h in vitro incubation with 0.125 mM palmitate significantly increased the mRNA expression of both chitinase 3-like protein 3 (Ym1) and arginase 1—two markers of alternative activation of immune cells [22], while LPS, known to induce classical activation of microglia, significantly reduced the expression of arginase 1 after 24 h (Figure 5F). No significant effect on the expression of arginase 1 could be detected after 2 h with LPS/palmitate (LPS: 103.7 ± 13.7 *vs.* LPS control: 106.7 ± 28.3/palmitate: 72 ± 11.6 *vs.* palmitate control 116.9 ± 30.6).

## Discussion

Here we show for the first time that BV-2 microglial cells express the proglucagon gene and secrete GLP-1, processes that are both enhanced by cAMP. We also show that this expression of proglucagon is downregulated, in conjunction with a reduced number of GLP-1-positive cells, in obese insulin-resistant animals *in vivo*, an effect mimicked in vitro in a diabetic milieu characterized by high levels of fatty acids. In line with our central finding that microglial cells secrete GLP-1, we also demonstrate that proglucagon mRNA expression and GLP-1-positive cells are detected in areas of the brain distinct from the brainstem and the NTS neurons—where proglucagon expressing neurons have previously been identified.

Throughout this study we have used *ob/ob* mice, a well-known and widely used animal model for obesity and insulin resistance. The *ob/ob* mice are characterized by high levels of free fatty acids and systemic inflammation [23-25]. The BV-2 cell line is commonly used since it offers the advantage of being comprised exclusively of microglial cells and at the same time retains a highly similar response pattern to primary microglia, especially in response to inflammatory stimuli [16].

We report here that acute adenylyl cyclase and PKA activation by forskolin increases proglucagon mRNA expression and GLP-1 secretion from microglia cells, showing that the proglucagon and GLP-1 levels can be manipulated by different stimuli. Further, we report that classical activation of these cells in vitro, induced by acute LPS treatment, reduces GLP-1 secretion, while acutely and transiently increasing proglucagon mRNA expression, indicating that the acute effect of LPS treatment initially interferes with the secretory mechanism. The specific steps impaired by LPS will be investigated in forthcoming studies.

Due to their insulin resistance, T2D patients often have elevated levels of plasma FFAs [26,27], and recent studies indicate an increased accumulation of fatty acids also in the CNS in diabetes [4]. Consequently, we aimed to evaluate whether high concentrations of fatty acids would alter the activation state and/or the proglucagon mRNA expression of microglial cells. Palmitate was chosen to evaluate the effect of high concentrations of FFAs relevant to T2D, as it is the most studied FFA in endocrine cell types. We used complexes where the molar ratio of FFA to albumin was 2:1, which is physiologically relevant. We report here that diabetic conditions simulated by palmitate treatment in vitro significantly reduce proglucagon mRNA expression in microglial cells.

In support of microglia being a source of the proglucagon mRNA expression detected in C57 black mice, the inhibitory effect of high levels of palmitate on proglucagon mRNA expression from microglial cells is in line with the downregulation of proglucagon gene expression in the *ob/ob* mice. However, GLP-1-positive cells are reduced in *ob/ob* mice, while GLP-1 secretion is not modified in palmitate-treated microglial cells. This discrepancy could stem from the relatively short treatment (24 h) with palmitate in vitro. It is possible that reduced GLP-1 secretion may come as a consequence of reduced proglucagon expression and be detectable at a later time point. Another possibility is that direct effects on GLP-1 secretion by the relatively short treatment with palmitate in vitro compensates for a reduced number of GLP-1-positive cells in the culture. Future studies will aim to investigate the relevance of these hypotheses. Noticeably, our results demonstrate that LPS and 24-h treatment of BV-2 cells with the saturated fatty acid palmitate will induce opposite effects, where palmitate treatment will reduce the mRNA expression and secretion of TNFα, whose induction is characteristic of a classical activation (LPS activation) of these immune competent cells, while inducing the alternative form of microglia activation. Differential activation in response to fatty acids and LPS has been reported for macrophages in other studies [28]. However, there are also studies supporting an increase in the mRNA expression and secretion of TNFα from monocytes in response to palmitate, although these studies generally use much higher concentrations of palmitate (0.5 mM) [29], which may not have the same clinical relevance as the concentration used in this study. Little *et al.* observed an increase of IL-8 and MCP-1 in palmitate-treated THP-1 cells [30], using the same concentration (0.125 mM) of palmitate as the present study. However, the cell type and the time differ from this study. Further, it is somewhat inconclusive what the elevated levels of IL-8 indicate since IL-8 has been reported to be associated with M2c (a subtype of the M2 phenotype)-activated macrophages, as reviewed by David and Kroner [31], as well as with the M1 phenotype. Moreover, it is well known that the microenvironment and previous insults to the cells can contribute to the activation state, thus making it difficult to pinpoint the exact effect of different stimuli. Further, our *in vivo* data showing reduced TNFα mRNA expression in an obese insulin-resistant mouse model further support the fact that high levels of fatty acids will reduce TNFα mRNA expression and secretion.

The positive correlation between TNFα and proglucagon mRNA expression in C57 black and *ob/ob* mice identified in this study, for example, decreased mRNA expression of an inflammatory cytokine and simultaneous decreased mRNA expression of a neuroprotective peptide, may have its biological importance in protecting the neurons from cytotoxic factors secreted to kill off invading pathogens. This is of course very speculative, but the possible biological relevance and importance of these novel findings should lead to a thorough assessment of the mechanisms.

The decrease of TNFα in *ob/ob* mice could stem from a change in microglia induced by fatty acids toward more alternatively activated microglia, as indicated by the present study, and thus lower levels of TNFα. However, this study does not investigate the effects of simulated hyperlipidemia in the context of LPS activation of microglia. It is possible, as indicated by studies in other tissues [32], that, in response to invading pathogens/infections and release of endotoxins, the high levels of fatty acids contribute to an enhanced/prolonged immune response that may have cytotoxic effects. To further investigate proglucagon mRNA expression and GLP-1 secretion in response to LPS/amyloid-β under hyperlipidemic conditions remains part of our future studies. In summary, this study provides novel information on GLP-1 production in the brain. We demonstrate that NTS neurons are not the only central source of GLP-1. Proglucagon is expressed in other areas of the brain and significantly reduced by (pre)diabetic conditions *in vivo* and in vitro. Specifically, this study demonstrates for the first time that microglia-derived cells secrete GLP-1 and that this secretion is regulated by the activation state of these cells, by forskolin leading to PKA stimulation and by LPS. One of our future research goals, in addition to further characterizing the mechanisms that regulate GLP-1 secretion from microglia, is to determine the effects of insulin-resistant conditions on GLP-1 secretion *in vivo* by comparing non-diabetic C57 black and obese *ob/ob* mice.

The novel information provided in this study and the central finding that microglia secrete the neuroprotective peptide GLP-1 may have very interesting pathogenic and therapeutic implications for the diabetes/neurodegenerative disease interface. Modulating the activation state of—and the GLP-1 secretion from—microglia may be a novel option for treating neurodegenerative diseases.

## Abbreviations

TNFα: Tumor necrosis factor α; GLP-1: Glucagon-like peptide-1; PKA: Protein kinase A; LPS: Lipopolysaccharide; T2D: Type 2 diabetes; Ym1: chitinase 3-like 3.

## Competing interests

The authors declare that they have no competing interests.

## Authors’ contributions

CK and LMT: Conceived the research plan, performed and designed the experiments, contributed to discussions, wrote the manuscript, performed analysis of data, and acquired and processed images and figures. CP: Participated in discussions and manuscript preparation and contributed to the research plan. KI: Provided expertise on AD and neuroinflammation, participated in manuscript preparation and discussions, and contributed to the research plan. ÅS: Provided expertise in T2D, participated in the manuscript preparation and discussions, provided the animal models used and contributed to the research plan. All authors read and approved the final manuscript.
